# Dietary Flavonoids in p53—Mediated Immune Dysfunctions Linking to Cancer Prevention

**DOI:** 10.3390/biomedicines8080286

**Published:** 2020-08-13

**Authors:** Shoib Sarwar Siddiqui, Sofia Rahman, H.P. Vasantha Rupasinghe, Cijo George Vazhappilly

**Affiliations:** 1 Department of Biotechnology, American University of Ras Al Khaimah, Ras Al Khaimah PO Box 10021, UAE; shoib.siddiqui@aurak.ac.ae; 2 School of Natural Sciences and Mathematics, The University of Texas at Dallas, Richardson, TX 75080, USA; sofiarahman0@gmail.com; 3 Department of Plant, Food, and Environmental Sciences, Faculty of Agriculture, Dalhousie University, Truro, NS B2N 5E3, Canada; vrupasinghe@dal.ca; 4 Department of Pathology, Faculty of Medicine, Dalhousie University, Halifax, NS B3H 4R2, Canada

**Keywords:** flavonoids, inflammation, p53, cancer prevention, Nrf2 pathway, anti-oxidant

## Abstract

The p53 protein plays a central role in mediating immune functioning and determines the fate of the cells. Its role as a tumor suppressor, and in transcriptional regulation and cytokine activity under stress conditions, is well defined. The wild type (WT) p53 functions as a guardian for the genome, while the mutant p53 has oncogenic roles. One of the ways that p53 combats carcinogenesis is by reducing inflammation. WT p53 functions as an anti-inflammatory molecule via cross-talk activity with multiple immunological pathways, such as the major histocompatibility complex I (MHCI) associated pathway, toll-like receptors (TLRs), and immune checkpoints. Due to the multifarious roles of p53 in cancer, it is a potent target for cancer immunotherapy. Plant flavonoids have been gaining recognition over the last two decades to use as a potential therapeutic regimen in ameliorating diseases. Recent studies have shown the ability of flavonoids to suppress chronic inflammation, specifically by modulating p53 responses. Further, the anti-oxidant Keap1/Nrf2/ARE pathway could play a crucial role in mitigating oxidative stress, leading to a reduction of chronic inflammation linked to the prevention of cancer. This review aims to discuss the pharmacological properties of plant flavonoids in response to various oxidative stresses and immune dysfunctions and analyzes the cross-talk between flavonoid-rich dietary intake for potential disease prevention.

## 1. Introduction

One of the hallmarks of cancer is genome instability due to DNA damage [1]. There are several genes implicated in modulating the pathways related to DNA damage and repair response [2]. An impetus to this field of research was provided by the discovery of the first tumor suppressor gene p53 in SV-transformed cells in 1979 [3]. However, the history of p53 is rather interesting. Initially, it was observed that p53 could accumulate in cancer cells and a knockdown of the gene led to the inhibition of cell proliferation. Thus, scientists presumed that it was an “oncogene.” The identification of new oncogenes was fascinating [4]. However, over the last four decades, p53 has evolved from the realization of a tumor suppressor protein to transcription factor, a regulator of metabolic pathways, a regulator of cytokine activity, and a drug target for cancer therapy [4]. Due to its role in the DNA damage and repair process, the tumor suppressor *p53* gene is often referred to as the “guardian of the genome” [5].

Several reports and observations showed the role of p53 as an oncogene. However, it was observed in many tumor models that the mouse *Trp53* is inactivated by the retroviral insertions pointing towards its potential role as a tumor suppressor gene [6,7]. This p53 conundrum took a turn when the cloned wild type (WT) p53 sequence was compared with the tumor expressing p53 and it was observed that the tumor-promoting activity of p53 is due to its mutant form found in cancer cells [4]. In an elegant study on colorectal cancer, it was shown that more than 50% of tumors had a loss of heterozygosity (LOH) at the p53 locus, thus providing further evidence of its role as a tumor suppressor [8]. Further experiments strengthen the notion that the wild type p53 can suppress oncogenic transformation [9]. The analysis of p53 knockout mice was interesting. The mice were developmentally normal but developed primary lymphomas and sarcomas at around 6 to 9 months of age [10]. In human patients, the early clues came in the 1960s from the group of people who were highly prone to develop cancer due to a rare autosomal mutation. This syndrome was called Li-Fraumeni syndrome (LFS) [11,12]. Later, the specific mutation was identified as a germline mutation in *TP53* [13,14].

### 1.1. p53 as a Transcription Factor

p53 acts as a transcription regulator under stress conditions. After the acceptance of p53 as a tumor suppressor, several studies were published in the early 1990s leading to the role of p53 as a transcription factor [15,16,17]. Wild type p53 has been shown to have DNA binding ability, while its mutant counterpart, often found in cancer cells, does not have this ability [15,16,17]. Moreover, the effect of p53 as a transcription activator is direct, but the effect as a repressor is indirect [15,16,17]. Further, p53 regulates the transcription of genes that are important for apoptosis, cell cycle, and DNA repair machinery [4]. p53 functions as a pioneer transcription factor. The binding of p53 to nucleosomal DNA takes place through the linker DNAs. p53 also binds to histone protein through the N-terminal 1–93 amino acid region [18]. In a recent study, it was identified that the transcriptional regulation of porcine p53 is very similar to human p53 for a number of target genes, and, thus, it is inferred that pigs can be used to study human diseases [19]. The role of p53 as a transcription factor is also shown in several disease models. Human dihydroorotate dehydrogenase (DHODH) is an enzyme involved in de novo synthesis of pyrimidines. Tetrahydroindazoles (HZ) have been identified as potent inhibitors of DHODH. HZ analogs have been shown as promising agents against cancer progression. The cell-based reporter showed that HZ functions by activating the p53-dependent transcription activity [20]. The autosomal dominant polycystic kidney disease (ADPKD) is mainly caused by the mutations in the PKD1 or PKD2 genes. The recent studies showed that PKD1 gene expression is controlled by the overlapping but opposing transcriptional activity of Myc and p53 [21]. Esophageal Cancer-Related Gene 2 (ECRG2) is a tumor suppressor whose activity is highly regulated by the transcriptional activity of p53. It was identified for two p53 binding sites within the promoter of ECRG2, and thus its mRNA and protein expression is regulated [22].

### 1.2. p53 as a Regulator of Metabolic Pathways

One of the hallmarks of cancer is the shift to glycolysis as the predominant metabolic pathway for the generation of energy, despite oxidative phosphorylation being an efficient way to produce adenosine triphosphate (ATP). This is referred to as the “Warburg effect.” Due to this, cancer cells utilize a higher amount of glucose compared to normal cells. Both the oncogenes and tumor suppressor genes have been shown to play a pivotal role in the regulation of this phenomenon. p53 is one of the factors that reduce the utilization of glycolysis and enhance the oxidative phosphorylation of cancer cells [23]. Thus, p53 plays a vital role in this metabolic pathway, which is also a hallmark for cancer cells [1]. p53, together with other tumor suppressor genes, carries out the function of immunological homeostasis. Moreover, the transcriptional activity of p53 regulates the expression and signaling of a number of cytokines and some of these cytokines activate p53 expression suggesting a positive feedback loop. In addition, the immune checkpoint regulators, such as program cell death protein 1 (PD1) and program cell death ligand 1 (PDL1), also cross-talk with p53 and thus modulate the overall immune responses [24].

### 1.3. p53 as a Drug Target for Cancer

As discussed previously, more than 50% of all cancer has a p53 mutation [25]. p53 is important for homeostatic signaling in cell proliferation, cell cycle regulation, apoptosis, senescence, and inflammation, and a mutation in this crucial protein leads to malignancy. The most common mutation in p53 is a single amino acid substitution, which leads to the loss of DNA binding function and misfolding of the protein. There has been a thrust to develop drugs that can restore the WT activity in the missense mutant proteins. This restoration of WT activity in mutant p53 is often referred to as a “reactivating mutant” [26]. Although many such drug candidates have been developed, most of them failed during the early developmental phase [26]. The reactivation of p53 is a major pharmacological intervention and has been suggested to have promising effects for cancer therapy. PhiKan083, also known as PK083, has been shown to reactivate p53 mutants under pre-clinical settings. Using virtual screening and validation on different p53 mutants, it was shown that Y220C and Y220S are the targets of PK083 [27]. In another study with virtual screening on natural compounds targeting the Loop1/Sheet 3 (L1/S3) of WT-p53, a drug candidate torilin was identified. Torilin not only improved p53 activity but also enhanced protein expression of p21. This ultimately led to the suppression of HCT116 cancer cell growth [28]. It is noteworthy that around 80% of triple-negative breast cancer (TNBC) patients have a mutation in p53. Therefore, reactivating p53 mutants by drugs can be prolific for clinical intervention. One such drug recently studied for TNBC is 2-sulfonylpyrimidine compound, PK11007. PK11007 targets the apoptotic pathway of cell death in TNBC cell lines [29]. These recent studies highlighted the role of p53 as an attractive drug target for cancer therapy.

## 2. p53-Mediated Inflammatory Response and Immune Signaling

The role of inflammation in the promotion of cancer is well defined and is considered as an important hallmark of the disease [1]. Cancer leads to inflammation and in many instances, chronic inflammation leads to cancer [30]. The oxidative stress induced by reactive oxygen and nitrogen species [31] and viral and bacterial infection is associated with cancer risk [32]. How p53 is involved in cancer-related inflammation in the tumor microenvironment is an interesting question. p53 acts as a suppressor of inflammation, but proof came from the p53 knockout mouse, which itself led to chronic inflammation in mice, but not sufficient enough to cause cancer. It was observed that many of the mice died before the progression of the tumor due to chronic inflammation [10,33]. In an experimental autoimmune encephalomyelitis (EAE) model, p53 knockout mice showed severe inflammation in the central nervous system (CNS) [34]. In a collagen-induced arthritis (CIA) model, p53 knock out mice developed more severe arthritis symptoms compared to WT DBA/1 mice [35]. This is further proof for the role of p53 as an anti-inflammatory target. Using an azoxymethane (AOM)-induced colon cancer model in a conditional knockout mouse of p53, it was shown that the loss of p53 in stem cells leads to tumor formation with a combinational effect of DNA damage and chronic inflammation [36].

Further, to identify the functioning of p53 as an anti-inflammatory agent, several studies have been carried out and linked with the transcription factor nuclear factor-κB (NF-κB). p53 suppresses the activity of NF-κB by inhibiting the transcriptional activity of p65 or suppressing the activation of p65 by IκB kinase (IKK) and proteasomal degradation of IκB. It is noteworthy that IκB acts as an inhibitor of NF-κB [37,38]. In the studies pertaining to gastric cancer caused by *Helicobacter Pylori*, it has been shown that the virulence factor cytotoxin-associated gene A (CagA) activates NF-κB and induces inflammation in gastric epithelial cells [39,40,41]. This activation of NF-κB further enhances the expression of activation-induced cytidine deaminase (AICDA), which promotes cancer by incorporating *TP53* mutations [42]. In addition, the inflammatory bowel diseases are characterized by the infiltration of neutrophils that are rich sources of free radical species. These free radicals have been shown to promote cancer progression by incorporating mutations in the *TP53* gene [39,43,44].

### 2.1. Cross-Talk between p53 and MHCI Pathway

The major histocompatibility complex (MHC), derived the name from its discovery with the research on tissue compatibility upon transplantation. These are an important class of gene complexes that play a pivotal role in recognition of foreign antigens. Being expressed on the cell surface, they interact with T-cell receptors (TCR) and pose an immune response against the endogenous or exogenous antigens [45]. MHCI is expressed on the surface of all nucleated cells (epithelial or fibroblasts) and is involved in showcasing the intracellular proteins on the cell surface in the form of short peptides. The processing and transport of these small peptides is a complex process that involves the interplay of several proteins, including transporter associated with antigen processing 1 (TAP1) and TAP2. It has been shown previously that one of the alleles of MHCI, human leukocyte antigen B7, can be transcriptionally repressed by the tumor suppressor gene p53 [46]. Several other studies pointed out that p53 can promote the processing of peptides inside the cell and modulate the surface expression of MHC1 [47,48]. In addition to these changes, the transcriptional activity of p53 can upregulate the expression of TAP1 (a protein involved in the transport of peptides toMHC1).

Moreover, p53 also upregulates the expression of ERAP1 (endoplasmic reticulum aminopeptidase 1), which leads to enhanced expression of MHCI on the cell surface [47,48]. The cumulative effect of this p53 processing resulted in an enhanced immune response against cancer. Interestingly, the mutant p53 has lost the activity of all these processes, which emphasizes the dysregulated immune function and oncogenic role of mutant p53. On the contrary, it has been observed that the loss of function of important genes in the MHCI processing pathway, such as β2 microglobulin and TAP1, also leads to a reduction in the function of p53, proposing a cross-talk between MHCI and the p53 pathway [39]. In a recent study, it was shown that the lack of p53 in medulloblastoma leads to the loss of MHCI expression and resistance to immune rejection. The mechanistic study showed that this is due to the loss of expression of TAP1 and ERAP1, but tumor necrosis factor factor-alpha (TNF-α) agonists can rescue the expression of these proteins. Moreover, in vivo studies also supported the notion that the p53 null state plays a pivotal role in immune evasion, and TNF can reverse this phenotype [49].

### 2.2. Cross-Talk between p53 and Immune Checkpoints

To mount an immune response, the MHCI showcase the peptide to the surface of the TCR of T cells. Once activated, T cells start to express PD1 that react with PD-L1. This PD1/PDL1 axis is instrumental in maintaining the protective immunity and homeostatic condition. Beside PD1/PDL1, a flurry of other receptor–ligand complexes keep the immune system in check. These receptor–ligand complexes are called immune checkpoints [50]. These immune checkpoints are vital for maintaining the physiological resting state of immune cells (T cells). However, in the case of cancer progression, cancer cells hijack this process and engage T cells for immune evasion. The overexpression of PDL1 ligands on their surface of cancer cells to engage with PD1 of T cells is well recognized. This engagement of PD1 with PDL1 ultimately leads to immunosuppression [51]. Targeting this pathway is considered as a hotspot for cancer immunotherapy. It was identified that miR-34a, which acts as a transcriptional target of p53, is actually a repressor of PDL1. Thus, p53/miR-34a/PDL1 can be a suitable target for cancer immunotherapy [52]. A loss of p53 activity by a mutation in human lung cancer has been shown to upregulate PD-L1 expression. Thus it will be instrumental in predicting the patient pool that may or may not be responsive to checkpoint inhibitors targeting the PD1/PDL1 axis [53].

### 2.3. Cross-Talk between p53 and TLRs

Toll-like receptors (TLRs) are the receptors that detect pathogen-associated molecular patterns (PAMPs), present mostly on the microbial surfaces [54]. Many TLRs were also found on the surface of cancer cells as well. TLR signaling on cancer cells mediates a number of functions that trigger cancer progression. Therefore, TLRs are considered as another attractive target for cancer immunotherapy [55]. It was shown that p53 could act as a transcriptional activator of TLR3 that can trigger agonist-induced apoptosis in cancer cells [56]. There is a cross-talk between the target genes of p53 and TLRs, and they together mediate the downstream signaling [57]. In the patients with mutant p53, the TLR4 expression inversely correlates with the survival time, and in the WT p53 patients, the TLR4 expression correlates directly with the survival time [58]. TLR8 is a target of p53 where its expression was increased due to a single nucleotide polymorphism (SNP) (rs3761624) in the TLR8 promoter [59]. In another study, a comparison of the expression of TLR1-TLR10, p53, and NF-κB in patients of oral lichenoid disease (OLD) with healthy individuals was interpreted. It was found that all TLRs and p53 were increased in OLD versus healthy individuals in all the layers. Moreover, a positive correlation was obtained between the expression of TLR5, NF-κB, and p53 in the intermediate layer in OLD patients [60]. Thus, there is a cross-talk between TLRs and p53 in the pathology of OLD. TLR4 is important in vascular inflammation, atherosclerosis, and diabetes. In the study to identify the role of palmitate-triggered apoptosis in the activation of TLR4 associated pathways, it was found that the palmitate promoted apoptosis in vascular smooth muscle cells (VSMC) and significantly enhanced the expression of p53. The knockdown of TLR4 led to a reduction in the expression of p53 in this model, directing towards the role of the TLR4/p53 axis in atherosclerosis [61]. Further, imiquimod (IMQ) is a synthetic TLR7 ligand used for the treatment of basal cell carcinoma (BCC). A thorough study was carried out in order to understand the role of p53 in IMQ triggered cell death in the skin cancer model. It was identified that IMQ could lead to the upregulation of p53 expression, its phosphorylation, and translocation in a TLR7/8-independent manner [62].

## 3. p53 in the Tumor Microenvironment and Cancer Immunotherapy

**Figure 1:**
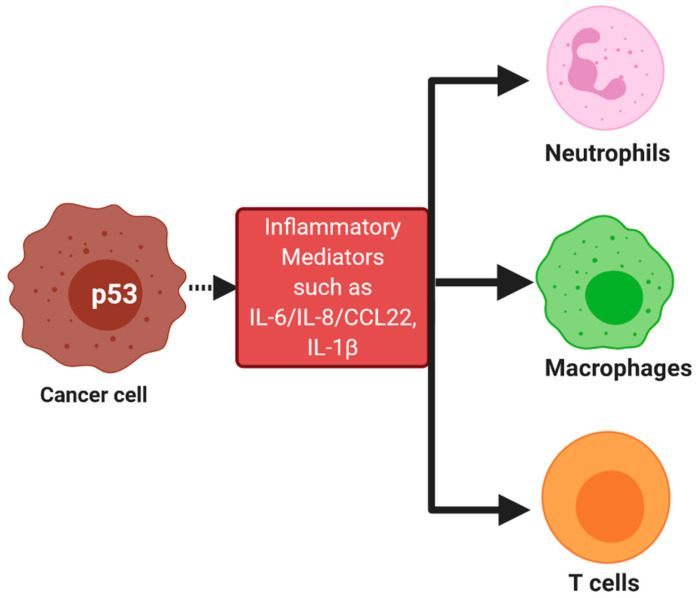
Infiltration of immune cells due to activation of p53: The activation of p53 in cancer cells can be due to many factors such as TLR5-flagellin interaction. Due to this activation of p53, the inflammatory mediators are released, which leads to the infiltration of immune cells in the tumor microenvironment. These immune cells include neutrophils, macrophages, and T cells.

The tumor microenvironment is very important in determining the fate of the tumor and its progression. One of the factors that shape the tumor microenvironment is p53. It has recently been shown that a loss of p53 can dramatically affect the immune cell composition of tumors [63,64] (Figure 1). The tumor-promoting macrophages are recruited in breast cancer, prostate cancer, and ovarian cancer with the loss of p53 [63,64,65]. On the other hand, p53 loss increases the response of tumor-associated macrophages (TAMs) to a variety of tumor types such as lung, pancreatic, ovarian, and carcinogen-induced skin cancers [65,66]. In the breast cancer model, the loss of p53 has been shown to involve the malfunctioning of the WNT pathway and infiltration of neutrophils, which supports cancer progression [63]. The infiltration of monocytes in the ascites of ovarian cancer is observed in p53 null state, probably due to the involvement of the C-C motif chemokine ligand 2 (CCL2) [65]. In p53 null tumors, the infiltration of tumor-suppressive myeloid CD11b^+^ cells and Treg was observed. The expression of C-C chemokine receptor type 2 (CCR2) associated chemokine and macrophage colony-stimulating factor (M-CSF) were responsible for blunting the immune response by targeting T helper and T cytotoxic cells [67]. Thus, tumor cells get the advantage of manipulating the immune microenvironment by the loss of p53 or the accumulation of mutations in p53 [39].

### p53 as a Target for Cancer Immunotherapy

Since p53 is highly regarded as a mutated gene in tumors, it can act as a target for cancer immunotherapy. Due to the intracellular localization of p53, targeting through antibodies was not possible. However, in a recent study, a novel antibody T1-116C was generated, which acts as a TCR mimic. This antibody recognizes several cancer types without recognizing normal blood cells. In the breast cancer xenograft model, it was showed to significantly reduce tumor growth [68]. A similar approach with affinity matured human antibody, P1C1TM, was shown to be effective in reducing the tumor growth both in vitro and in vivo [69]. In a recent report, the role of p53 in systemic inflammation was deciphered. In the breast cancer models, a loss of p53 in cancer cells led to an increase in WNT ligands, which triggered the macrophages to produce interleukin 1 beta (IL-1β) that ultimately led to systemic inflammation. Thus, this study was a direct demonstration of p53 as a mediator of inflammation, neutrophilia, and metastasis [63].

Constitutive activation of the signal transducer and activator of transcription 3 (STAT3) pathway is an important factor in determining the tumor stroma and hence considered as a potential target for cancer immunotherapy [70]. It is known that the loss of p53 leads to constitutive activation of the STAT3 pathway in pancreatic cancer. Moreover, the mutations in *p53* genes in human pancreatic cancer correlate with the poor prognosis and survival time [71]. In addition, the ablation of p53 also leads to enhanced reactive oxygen species (ROS) production that inhibits Src homology region 2 (SHP2) activation and triggers STAT3 expression [71]. In a phosphatase and tensin homolog (PTEN) knockout mouse and embryonic fibroblasts, p53 deficiency led to an increase in STAT3-myc signaling [72]. STAT3 is negatively regulated by suppressors of cytokine signaling 1 (SOCS1) protein that binds to the N-terminal domain of p53, leading to cell cycle arrest and senescence [73]. One of the mutants of p53, R175H, has been shown to enhance NF-κB signaling and increased localization to one of its subunits p65 [74]. Some of the mutants of p53 proteins also directly interact with p65 leading to increasing NF-κB transcriptional activity [75]. It has shown that the mutant p53 protein inhibits the expression of secreted interleukin-1 receptor antagonist (sIL-1Ra) and, thus, promotes malignancy. sIL-1Ra is a target gene for mutp53 and thus, a potential therapeutic target for combating tumor growth [76]. *TP53* gene mutation is frequent in hepatocellular carcinoma (HCC). As discussed earlier, p53/mir-34a/PD-L1 and TLRs can also be considered as targets for cancer immunotherapy.

## 4. Chronic Inflammation and Cancer

**Figure 2:**
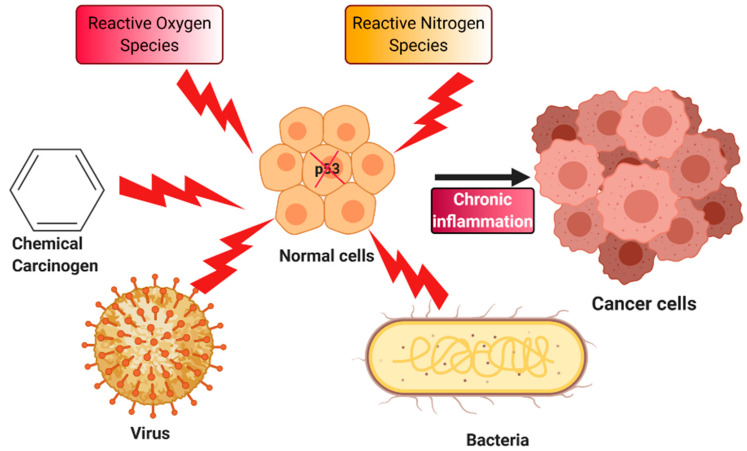
Chronic inflammation-induced carcinogenesis: The process of carcinogenesis sometimes involves chronic inflammation induced by chemical carcinogens, reactive oxygen and nitrogen species, viruses, and bacteria. All these agents trigger mutation in the *p53* gene that ultimately leads to the transformation of normal cells into cancer cells.

Invasion of pathogens, increased toxicity in the cells, or tissue injuries are some of the traumas that the human body combats with the progression of inflammation. Activation of inflammation is initiated by immune cells such as mast cells and macrophages, and progressively results in the production of inflammatory mediators, such as pro-inflammatory cytokines and chemokines, whose roles are to amplify the inflammatory response in order to resolve the trauma [77]. Collectively, these inflammatory reactions lead to the release of ROS and reactive nitrogen species (RNS), in attempts to aim for tissue repairing by cellular and immune changes concluding in the amendments of the site of injured cells by cell proliferation [78]. Thus, it can be noted that the purpose of inflammation is to solve the cellular impairments and restore cellular homeostasis. Similar to every homeostatic maintenance behavior, the initial process should be able to shut down after the resolution of the initiator. Therefore, in this case, after the restoration of the damaged tissue by the regular rate of cell growth, the inflammatory processes should be able to suspend and establish inflammation resolution through multiple mechanisms. One for instance, is moving from pro-inflammatory to anti-inflammatory reactions by the intervention of the released ROS [79]. However, if the ability of the inflammation is not resolvable, it results in the building up of the inflammatory response products, and transformation from acute inflammation to prolonged chronic inflammation. Another source of chronic inflammation has been identified as when the acute inflammatory processes are unable to rectify the cellular damages, thus becoming uncontrolled and chronic, which further play a role in the development of chronic inflammatory diseases [80]. In chronic inflammation, the immune cells maintain a pro-inflammatory nature, aggravating the by-products released by the immune cells, which co-results in continuous uncontrolled cell proliferation. ROS and RNS are two such by-products that are considered to aid in chronic inflammation-related cancer [81]. Additionally, ROS and RNS are sources of DNA mutations, as they generate peroxynitrite. Peroxynitrite has been defined to possess mutagenic properties such as DNA strand lesions and nitration of the guanine bases [82]. The 8-oxo-7,8-dihydro-2′-deoxyguanosine (8-oxodG) and 8-nitroguanine are the examples of the DNA breakages formed by the ROS and RNS, which are known to exhibit DNA mutations and have been indicated in inflammation-associated cancer [83]. Furthermore, experimentations performed over two decades revealed that when human bronchial epithelial cells are exposed to high levels of nitric oxide (representative sources of RNS) which introduced superoxide ions, this resulted in transition mutations of the C:G pair to a T:A pair at the 248th codon of the *p53* gene [84]. Whereas, it can be observed that the chronic inflammation is leading the pathway towards carcinogenesis (Figure 2). It has been stated earlier in multiple literatures that at every stage of carcinogenesis, like cell multiplications, inhibition of senescence, resistance against anti-growth signals, and metastasis, chronic inflammation is the promoter for cancer [78,81]. Along with being mentioned as a promoter of cancer, it has also been made evident that in cells with chronic inflammation, there is a depravity of cell repair and cell death mechanisms, and instead of a promotion of abnormal cell proliferation [85].

Several immunomodulatory protein families are also implicated in inflammation. One such protein family is Siglecs [86]. Siglecs are receptors that are expressed on the surface of white blood cells and bind to sialic acids (Sia), a nine carbon atom monosaccharide [86,87,88]. Most members of this family carry an inhibitory motif and lead to the suppression of immune response [86,87,88]. Siglecs have been implicated in immunological response in many physiological and pathological conditions, including cancer [89,90,91,92,93,94]. In the case of a tumor, the cancer cells start to overexpress sialic acid, and the engagement of Siglecs on immune cells leads to immunoevasion and ultimately enhanced tumor growth [90,91]. A few members of the Siglec family are also activating in nature, and the engagement of these receptors leads to enhanced immune response and reduced tumor growth [92,95]. As priorly mentioned, oxidative stresses contributed by the inflammatory mediators and their by-products, seem to act as crucial protagonists in the development of carcinogenesis. Once inflammation-mediated cancer is established, it gears in pathways to strive onwards with the tumorigenic activities. One such pathway is the nuclear factor erythroid 2–related factor 2 (Nrf2) pathway that regulates the cancerous development.

### NRF2 Pathway and Its Role in Cancer

At normal conditions, Nrf2 acts as the transcription factor that binds the anti-oxidant response element (ARE) that initiates the expression of anti-oxidant and cytoprotective enzymes. The Nrf2 factor is maintained at a dormant phase by its negative regulator: the kelch-like ECH associated protein (Keap1)-Cullin 3 based ligase (CUL3) complex [96]. The Keap1-CUL3 complex sustains lower levels of Nrf2 by ubiquitination and proteasomal degradation, keeping it in a silenced phase [97]. Normally, Nrf2 is induced with increased oxidative stress, where it detaches from the Keap1-CUL3 complex, halting their antagonistic activity, enters the nucleus, binds to the ARE, and consequently expresses greater than 200 genes for anti-oxidative pathways [98]. However, in chronic inflammation-induced oxidative stress, the Nrf2 can escape its negative regulation performed by Keap1 by direct cause of increased oxidative stress that results in the upregulation of the p6 protein: an activator of Nrf2, and subsequently dismantles autophagocytosis of the p6 protein [99]. Nrf2 activity has been known to be exemplified by inhibiting the activities of Keap1 directly, such as p21Cip/WAF1, a competitive inhibitor of Keap1, inhibiting it from attaching to the DLG functional domain of Nrf2, thereby disrupting its ability of Nrf2 degradation [100]. Thus, it can be concluded that in chronic oxidative stresses, there is a hyper-regulation of Nrf2 activation. Similarly, when dealing with chronic inflammation-mediated cancers, there has been evidence that the Nrf2 factors are overexpressed, which could be due to mutations, changes in DNA methylation patterns, and protein alterations [101]. Carcinogenic promoting properties are harnessed by the Nrf2 in cancer cells, playing vicious roles in promoting cell proliferation, inhibiting apoptosis, and strengthening its resistance to chemo/radiotherapy [102]. One other interesting property imparted by Nrf2 to cancerous cells is drug resistance. It has been found in doxorubicin-resistant human ovarian cancer cells, the upregulation of Nrf2 resonates with its character of resistance [103]. Moreover, it has been described as a promoter of all the necessary key hallmarks needed to establish cancer, from increasing protein synthesis and expression of cell division genes to stimulating mRNA translations in support of cell proliferation [96].

## 5. Oxidative Stress, p53, and Inflammation

Oxidative stress is referred to as a state of imbalanced equilibrium between the generation of ROS with the anti-oxidant system of the body [104]. A balanced redox mechanism is necessary for maintaining genomic integrity. Excessive oxidative stress can cause immune dysfunction and, thereby, induce an inflammatory response. For instance, the immune cells, such as T lymphocyte activation, were found to be suppressed by excessive ROS leading to inflammation and tumorous conditions. ROS, therefore, plays a dual role by either suppressing T lymphocyte activation or its intermediate level modulate T lymphocyte differentiation at the site of injury. Contrastingly, a persistent level of ROS enhances apoptosis in T cell lymphocytes by damaging DNA through a p53 pathway [105]. Several methods have been adopted to sensitize T lymphocytes to the site of injury and inflammation. Amongst these, the use of polyphenol such as resveratrol was found to boost T lymphocytes significantly in mice by enhancing CD86 and MHC-II antigens [106]. Another phytochemical, lycopene, alleviates oxidative injury in ruminant animals by inhibiting inflammatory cytokines and apoptosis. The anti-oxidant potential of lycopene ameliorates hydrogen peroxide (H_2_O_2_)-induced inflammation by regulating the Nrf2-ARE pathway in primary bMEC and MAC-T cells [107].

Further, in vivo studies have also shown a correlation between increased oxidative stress leading to neurobehavioral disorders. Buthionine sulfoximine (BSO)-induced oxidative stress in BTBR T^+^ tf/J (BTBR) mice have resulted in depletion of the native enzymatic anti-oxidant system, which has resulted in autism-like repetitive behaviors [108]. A systemic review also suggested that an imbalanced redox potential may contribute to neurodegenerative diseases by altering differentiation and the number of CD4^+^ T cell subpopulations [109]. Several reports indicated that oxidative stress-mediated inflammation could lead to cancer progression. Activation of transcription factors including NF-κB, AP-1, p53, HIF-1α, PPAR-γ, β-catenin/Wnt, and Nrf2 can transform normal cells into cancerous cells. Furthermore, a cross-talk between NF-κB activation and cell proliferation regulates the immunological and inflammatory response resulting in tumor conditions [110]. Such reports also indicated the possibility of using NF-κB as a prognostic marker to identify cancers, in particular, colon cancer [111]. It is noteworthy to observe that these transcriptional factors mediate p53-dependent fashion in response to an inflammatory condition.

Oxidative stress-induced p53 activation is common in inflammation and cancers. Activation of p53 primarily regulates the cell cycle, DNA repair, and apoptotic mechanisms [112]. Cancer cells exhibit higher ROS levels than normal cells since they are serving as an oncogenic agent with increased metabolism and mutation rates [113]. DNA damage caused by chemotherapeutic drugs, environmental and chemical toxins, radiations, and immunosuppressive drugs often elevates ROS levels, leading to p53 activation. Homeostatic regulation of ROS and p53 is necessary to determine the fate of normal tissue stem cells. Recent data have shown the direct relationship between ROS and p53 for the differentiation of stem cells. The lack of p53 functioning in the neural progenitor cells leads to increased ROS and premature differentiation [114]. Meanwhile, restoring ectopic expression of p53, and with anti-oxidants, enhances partial differentiation and stemness [115]. Many data have shown the dual mode of p53 functioning depending on the nature, duration, and intensity of oxidative stress imposed in different cell types. Hence, either anti-oxidant or pro-oxidant regulatory functioning of p53 is well known, depending on the type of cell burden. Activation of p53 by anti-oxidants imposes a protective mechanism against oxidative stress by ameliorating excessive ROS levels in the cells. Further, both endogenous and exogenous supply of anti-oxidants could play a role in inhibiting adhesion and invasion properties of cancer cells [116,117]. In contrast, pro-oxidants exert oxidative stress-induced p53 functioning leading to autophagy, caspase-mediated apoptosis, and necrotic cell death mechanisms [118,119]. A detailed analysis of anti-oxidant versus pro-oxidant effects on p53 mediated functioning is discussed elsewhere [116,120].

## 6. Plant Flavonoids as Therapeutic Agents

**Figure 3:**
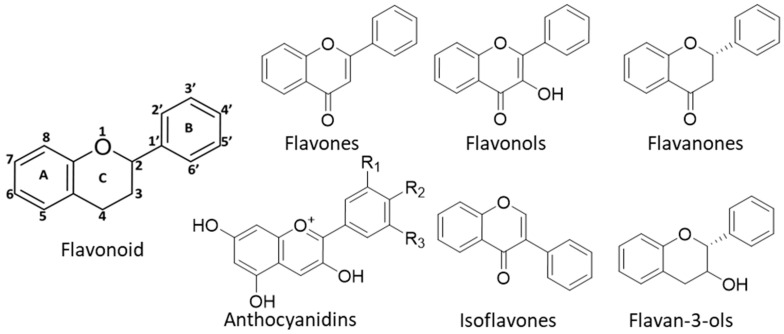
Basic structure of a flavonoid and its subclasses.

Over the last two decades, plant polyphenols, especially flavonoids, have gained significant attention in developing treatments for disorders such as Parkinson’s disease, inflammatory bowel disease, Alzheimer’s disease, obesity, cancer, and cardiovascular diseases (CVDs) [121,122]. A plethora of research has been done already to show how flavonoids influence these diseased conditions, mostly with their biochemical and pharmacological properties such as pro-oxidant and anti-oxidant potentials that correlates to their structure–activity relationship. Plant flavonoids share a basic structure of diphenyl propane in which two benzene rings are connected through a pyran ring. The subclasses of flavonoids such as flavones, flavonols, isoflavones, anthocyanin, flavan-3-ols, and flavanones differ by the attachment of carbon on the C ring to which the B ring is attached (Figure 3) [123]. Further, the degree of unsaturation and oxidation of the C ring contributes to the different classifications of flavonoids.

The structure-activity relationship of flavonoids majorly attributes to their observed clinical effects both in in vitro and in vivo studies. For instance, an anti-oxidant property of a flavonoid largely depends on the functional group it possesses and its spatial arrangement around the nucleus. The importance of the B ring and the hydroxyl group at position 3 often contributes to ROS scavenging potentials, as evident by the experimental and theoretical derivations. Furthermore, this spatial arrangement of the functional group and its substitution, along with the number of sugar moieties (glycosides) and hydroxyl groups, determines the mechanism of anti-oxidant potentials observed for several flavonoids [124,125]. Anti-oxidants exert various radical scavenging properties either by inhibiting chelated trace elements or enzymes that generate excessive ROS and/or by enhancing endogenous anti-oxidant enzymes such as glutathione peroxidase (GPx), catalase (CAT), and superoxide dismutase (SOD). In contrast, the pro-oxidant effect of flavonoids can result in preventing cancer through inhibiting the proliferation of tumor cells with apoptotic induction. Recent structure–activity studies have shown that the flavonoids possess significant anti-proliferative effects, especially in the presence of di-OH 3′, 4′, a double bond at C2-C3 and a carbonyl at the C4 position. However, flavonoids that possess functional chemical moiety at the C7-C8 position showed low to non-significant anti-proliferative effects [126]. These observations further warrant discussions on the importance of flavonoids in treating disease, especially inflammation and cancer, which are discussed below.

## 7. Flavonoids in Inflammation and Cancer Prevention

The p53-mediated pathway has been an attractive target for many of the flavonoids in regulating inflammation and cancers. Flavonoids regulate transcription factors such as NF-κB, Nrf2, and AP-1 in inflammation and DNA damage, cell cycle, and apoptosis in tumor cell proliferation. We have reviewed dietary flavonoids such as quercetin, luteolin, cyanidin, daidzein, and epigallocatechin gallate (EGCG) along with other flavonoids, which showed promising results in preclinical and clinical trials. While many reviews are available on discussing how different flavonoids can be used to induce apoptosis in cancer models, we have focused on flavonoids and their ability to prevent inflammation and cancers with a focus on p53-mediated mechanisms.

### 7.1. Quercetin

Quercetin, a flavonol found in vegetables and fruits, has been reported for various pharmacological properties that include scavenging ROS, inhibiting inflammation, and preventing cancer. One of the mechanisms by which quercetin exerts cytoprotective effects is through regulating MEK/ERK and Keap1/Nrf2/ARE pathways in inflammation [127]. An in vitro study has shown the protective effect of quercetin in cigarette smoke extract-induced inflammation by downregulating cytokine markers such as IL-1β, IL-6, and IL-8 in ARPE-19 cells. Pretreatment with quercetin further activates the Keap1/Nrf2/ARE-anti-oxidant pathway, which induces cellular defenses against various oxidative stress [128]. Recent reports have shown that p53 can activate the Keap1/Nrf2/ARE-anti-oxidant pathway and can act as the main regulator of the cells anti-oxidant response [129,130]. This effect of p53 largely depends on the intensity and degree of oxidative stress imposed. A lower level of oxidative stress has been correlated with increased anti-oxidant enzymes, such as SOD and CAT [131]. A flavonoid-rich extract from *Rosa laevigata* Michx fruit exerts neuroprotective effects by downregulating the levels of p-JNK, p-ERK, and p-p38 in MAPK pathways [132]. Furthermore, various in vivo studies in mice and rats have shown the downregulation of anti-inflammatory markers with increased anti-oxidant defense proteins (SOD, CAT, GPx) under oxidative stress-induced conditions, such as adipose hypertrophy, hepatocarcinogenesis, and obesity-induced skeletal muscle inflammation [133,134]. Further, quercetin significantly reduces adipocyte size and enhanced angiogenesis and adipogenesis by downregulating TNFα and HIF-1 α levels along with proteins such as TLR-4, CD68, MCP-1, and JNK [135].

Quercetin has been extensively studied to explore its mechanism of action in cancer prevention. Treatment with quercetin was reported to protect DNA damage induced by H_2_O_2_ in human Caco-2 cells. Quercetin decreases single-strand DNA breaks by enhancing the expression of human 8-oxoguanine DNA glycosylase and DNA repair mechanisms [136]. However, the detailed mechanism of p53 involvement in cancer prevention by quercetin remains unclear. It is believed that upon regulating p53 mechanisms, quercetin possesses a dual role and can have better control over cellular events to mitigate oxidative stress imposed by carcinogens through activating the Keap1/Nrf2/ARE anti-oxidant pathway and/or inducing apoptosis. In a recent study by Wang et al., quercetin was able to inhibit apoptosis by downregulating p53, Bax, and caspase-3 expression in ARPE-19 cells, and, thereby, it prevents high glucose-induced injury [137]. A similar kind of protective effect was also noted in the study by Darband et al., in which quercetin was able to inhibit oxidative DNA damage by activating the Nrf2 signaling pathway. Supplementation of quercetin (50 mg/kg) in rats reduces colon carcinogenesis induced by 1,2-dimethylhydrazine (DMH) [138]. In contrast, a study by Clemente-Soto et al., demonstrated the activation of p53 upon quercetin treatment in cervical cancer cells leading to apoptosis and DNA damage [139]. There are several reports available to demonstrate the similar functioning of p53-induced apoptosis, leading to cancer growth inhibition and prevention. This could be attributed to the multiple facts that the quercetin binding potentials to several receptors that may be important in the prevention of carcinogenesis, induces epigenetic modifiers, and interferes with enzymes such as native anti-oxidants, to elicit its protective mechanisms. Despite these properties, quercetin could sensitize and protect noncancerous cells during chemotherapy or radiotherapy when used in combination with existing classes of cancer chemotherapeutic-drugs [140].

### 7.2. Luteolin

Luteolin, a flavone found in many plants such as *Salvia tomentosa* Mill. (Balsamic sage) and *Glossogyne tenuifolia* possess anti-inflammatory effects via downregulating the NF-κB pathway. Luteolin was found to inhibit inflammation induced by infectious pathogens such as *Staphylococcus aureus* to cause mastitis. Treatment with luteolin reduces cytokines expression such as TNF-α, IL-1β, and IL-6 in a mouse model mimicking mastitis. Furthermore, luteolin suppressed the expression of IκBα and NF-κB, along with matrix metalloprotease-2 (MMP-2) and MMP-9, to render protection against inflammation [141]. A recent report showed the protective effect of luteolin against testicular deficits caused by lead acetate. Luteolin (50 mg/kg) treatment in male Wistar rats significantly activates the Nrf2/HO-1 pathway by increasing native anti-oxidant enzymes and inhibits inflammatory and apoptotic cascades [142]. These protective effects could relate to the anti-oxidant potential of luteolin [143] and it’s functioning on the p53 protein, as it is suppressed in *Staphylococcus aureus*-induced inflammation, preventing apoptosis [144]. Furthermore, a study by Li et al., proved the anti-oxidant nature of luteolin by activating the Nrf2 signaling pathway and NF-κB-mediated inflammatory responses in type 1 diabetes in mice. Modulation of these pathways reduces MMP protein expression and cellular hypertrophy [145]. These studies show that the protective effect of luteolin against inflammation largely depends on its anti-oxidant nature rather than its pro-oxidant effect, which inhibits different cancer cell proliferation [143].

Luteolin exerts its cancer-preventive properties by inhibiting DNA damage and activating anti-oxidant mechanisms in a p53 regulated manner. Combinational treatment of luteolin and EGCG showed translocation of p53 to the mitochondria, thereby regulating apoptosis [146]. This translocation of p53 is crucial and depends on the degree of stress burden imposed. The mitochondrial translocation occurs if the damage or stress is irreversible [147]. Further, in a study by Jiang et al., luteolin exerts its tumor-preventive potential by regulating the expression of microRNAs (miRNAs). Treatment in non-small cell lung cancer cell lines reduced cell proliferation and tumor inhibition in the H460 xenograft tumor model. The expression of an enhanced miR-34a-5p level was observed in tumor tissues, which resulted in a reduced level of MDM4 proteins. Furthermore, luteolin treatment enhanced p53 and p21 expression and reduced tumorigenesis [148]. The isolated compound luteolin from *Melissa officinalis* L, showed DNA damage protective effects induced by ultraviolet B radiation (UVB) in skin cells. Treatment with luteolin reduced DNA double strand breaks and the DNA damage response (DDR) mechanism in these cells [149]. Many studies have shown the upregulation of p53-mediated apoptosis for tumor inhibition in various cancer cell lines [150,151] and are not in the scope for this review.

### 7.3. EGCG

An anti-oxidant potent flavan-3-ol, EGCG, mostly seen in green tea polyphenols, showed anti-inflammatory effects under imposed oxidative stress conditions. EGCG exhibited an anti-inflammatory effect by inhibiting pro-inflammatory cytokines’, p53, NF-κB, TLRs, and STAT3 expression. In a recent study by Wang et al., rats were administrated with EGCG (50 mg/kg) and analyzed for parameters for anxiety-like behavior and myocardial infarction. ELISA and PCR studies have shown a significant reduction of IL-6 along with STAT3 expression. Further, inhibition of apoptosis cascades (caspase expression) was observed in rats. Hence, the intake of EGCG reversed anxiety-like behavior and prevented inflammation [152]. Similar observations were made by Ren et al., wherein lipopolysaccharides (LPS)-induced retinal inflammation in rats was attenuated by green tea extract (containing EGCG) through inhibiting phosphorylation of STAT3 and NF-κB along with pro-inflammatory cytokines [153]. Further reports have shown a strong association between EGCG and TLRs, which play an important role in the host immune system. TLR4 has a strong relation with inflammatory response and cancer progression [154]. It is interesting to observe that the p53-mediated differential expression of TLR4 depends on the context of the cells. In p53 wild type cells, the activation of TLR4 results in type-I IFN (IFN-γ) secretion, resulting in p21 mediated cell cycle arrest. Whereas in p53 mutant cells, the activation of TLR4 promotes cancer [58]. EGCG was shown to inhibit TLR4 expression in in vitro and in vivo studies [155,156]. However, EGCG’s direct involvement in p53-mediated regulation of TLR4 is not fully understood.

EGCG, being an active anti-oxidant, protects DNA damage mediated cell death in many cells. As observed for other flavonoids, EGCG exerts its preventive potentials by modulating Nrf2 and JAK/STAT pathways in testicular ischemia reperfusion injury-induced oxidative damage. Pre-perfusion treatment with EGCG prevented phosphorylation of JAK2, STAT3, and STAT1 along with apoptotic markers [157]. Likewise, EGCG prevents apoptosis and astrogliosis induced by acrylamide in rats. Pretreatment in rats with EGCG showed reduced DNA fragmentation, Bax, Bcl-2, caspase 3, and cytochrome c expression. Further, EGCG enhanced native anti-oxidant enzymes and glutathione levels to mitigate excessive ROS production [158]. EGCG was also shown to modulate DNA methylation processes, especially DNA methyltransferase 1, thereby rendering DNA protection [159]. Genomic stability of the cells can be safeguarded against various carcinogens by consuming flavonoids. Detailed mechanisms on DNA damage response and prevention are discussed elsewhere [160,161].

### 7.4. Cyanidin

Cyanidin, an anthocyanidin, found in plant pigments and berries [162] including grapes, bilberry, and blackberry, has been found to possess anti-inflammatory effects under oxidative stress conditions. Cyanidin-3-glucoside (C3G) is a naturally occurring anthocyanin in plants and cyanidin is the aglycone of C3G. Both C3G and cyanidin have shown significant anti-inflammatory effects against 2, 4, 6-trinitrobenzenesulfonic acid (TNBS)-induced colitis in mice. Administration of 200 μmol/kg in mice reduces inflammatory markers (TNF-α, IL-1β, IL-6, and interferon-γ) and thereby protecting the intestinal barrier [163]. Similarly, in vitro effects have been studied on Caco-2 cells, which showed inhibition in the disruption of intestinal barrier dysfunction. Further, C3G was found to improve diabetic conditions, especially diabetic nephropathy (DN). Eight weeks supplementation of 10–20 mg/kg of C3G in rats improves native oxidative enzymes and modulate the TGF-β1/Smad2/3 pathway to render renal protection [164]. This effect was further improved by encapsulating C3G in chitosan nanoparticles for targeted drug delivery and resulted in p53 mediated protection in mice. C3G downregulated p53-mediated apoptosis in mice with a balanced B-cell lymphoma-2/leukemia-2 ratio [165].

Further, C3G rendered DNA damage protection in BEAS-2B and HaCaT cells in vitro from oxidative stress imposed by nitrosamine, 4-(methylnitrosamino)-1-(3-pyridyl)-1-butanone (NNK) and UVB, respectively. In both studies, the degree of DNA damage was decreased by modulating ATM/ATR pathways, which is mediated by p53 expression [166,167]. Treatment with C3G restores the Bcl-2 expression in HaCaT cells and inhibits caspase-3 activation. Our previous study on apple peel flavonoid fraction (AF4) containing C3G as the main constituent, showed a DNA protective effect on NNK, methotrexate (MTX), and cisplatin-induced toxicity in BEAS-2B cells. Pretreatment with AF4 reduced the ROS level with an increase in anti-oxidant enzymes. Further, AF4 reduces DNA damage and enhanced protection against carcinogens by facilitating DNA repair mechanisms [168]. A study by Kaewmool et al., showed both anti-inflammatory and apoptotic effects of C3G in PC-12 cells. Anti-inflammation was mediated by decreased expression of IL-1β and IL-6 along with inducible nitric oxide synthase (iNOS) and Cox2 expression, while the apoptotic effect was mediated by a caspase-3 induced mechanism [169].

### 7.5. Daidzein

**Figure 4:**
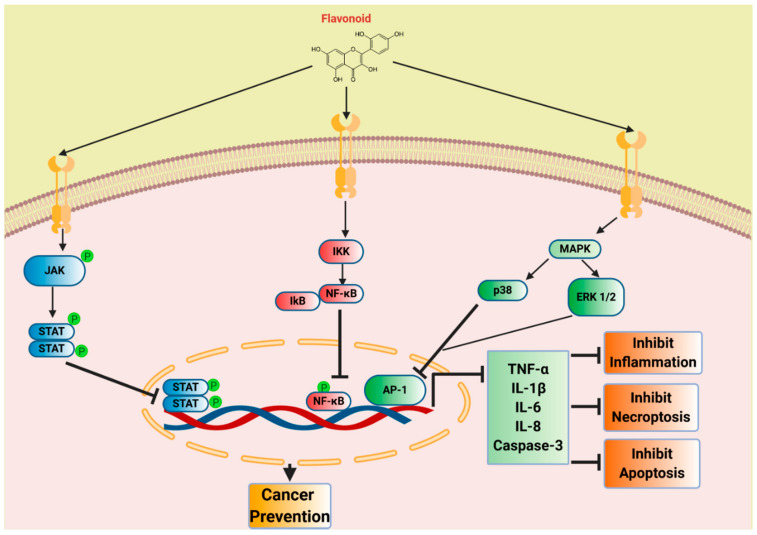
Flavonoids inhibiting inflammatory and cancer markers: The flavonoids can modulate several receptors and their corresponding signaling cascades. The flavonoids exert its effects mainly through JAK/STAT, IKK/NF-κB, and MAPK pathways leading to inhibition of pro-inflammatory, pro-apoptotic, and pro-necroptosis markers. The effector molecules that are downregulated include cytokines and cell death markers such as IL-1β, TNF-α, IL-6, IL-8, caspase-3, etc. These cellular events ultimately lead to immune regulation and cancer prevention.

Daidzein, an isoflavone, found in soybeans and soy products, is known to possess anti-inflammatory and cancer-preventive properties by inhibiting various inflammatory cytokines and cell death markers, respectively. Daidzein was found to inhibit the nephrotoxicity posed by cisplatin-induced oxidative stress in proximal tubular cells of mice. Treatment with daidzein inhibits the expression of TNFα, IL-10, and IL-18 along with enhanced anti-oxidant defense enzymes (SOD and GPx) activity. Further, daidzein reduces kidney injury markers (NGAL, BUN, creatinine, and KIM-1) and cell death mechanisms (caspase-3/7) [170]. Daidzein also improves the adverse effect of adipose inflammation by regulating the proliferator-activated receptor γ (PPARγ) in 3T3-L1 adipocytes. A decreased expression of monocyte chemoattractant protein-1 (MCP-1) and TNF-α was noted in adipose tissue of daidzein-fed mice [171]. Similar effects were observed by Feng et al., wherein daidzein treatment in rats inhibited TLR4 and NF-κB activation leading to the prevention of inflammation [172]. Furthermore, the synergic effect of daidzein and genistein exerts photoprotective potentials by regulating growth arrest and DNA damage (gadd45) genes, to decrease DNA damage and enhances DNA repair process in UV-induced photodamage [173,174]. The possible mechanisms by which flavonoids exert their anti-inflammatory and cancer-preventive properties are illustrated in Figure 4. Other flavonoids and their anti-inflammatory and cancer-prevention properties are summarized in Table 1.

## 8. Flavonoids and Dietary Intake

Recent epidemiological, cross-sectional and short-term randomized controlled studies have proved a direct relationship between flavonoid-rich diet intake and health benefits resulting in preventing various diseases. Flavonoids, with numerous potentials, as discussed above, improve life-threatening conditions, particularly cancer and CVDs. This direct correlation is based on several factors such as type/composition of flavonoid(s), their bioavailability after consumption, and the epidemiological region.

For instance, the average intake of flavan-3-ol among Europeans ranges between 77 mg/day to 182 mg/day probably because of the high consumption of tea, at least in the UK [201]. A large portion of ingested flavan-3-ol remained in the large intestine as they are not readily absorbed by phase II conjugating enzymes. This availability of flavan-3-ol could benefit microbiota in the colon region to metabolize further into monomers and contribute to an enhanced immune system [202,203]. In other countries of Europe (except the UK), a rich flavan-3-ol diet was mostly observed because of the consumption of non-citrus fruits (apples/pears) [201]. A meta-analysis of epidemiologic studies has shown a positive correlation between the consumption of flavan-3-ol and reduced cancer. The higher consumption of flavan-3-ol reduces rectal, oropharyngeal, breast, and laryngeal cancers [204]. In contrast, a study among 38,408 women aged ≥45 y with a frequent diet enriched with flavonols did not correlate with cancer risk prevention. The best explanation for the discrepancy could be due to a relatively low range of flavonoid intake combined with populations free of CVDs and cancers. However, continuous efforts have been made to overcome such issues as well as lipophilic nature, low solubility, and variable bioavailability of flavonoids by introducing different formulations to nanocrystals technology [205].

Another intake of flavonoids, such as anthocyanins and flavanones, exhibited a lower risk of CVDs in both women and men. A study conducted in 43,880 healthy men who have the habit of consuming a higher anthocyanin intake showed a lower risk of developing ischemic stroke over a period of 24 y [206]. Various studies have shown that the anthocyanins exert healthy beneficial effects on LDL-cholesterol level, which is mediated by cholesterol efflux capacity [207]. In a randomized controlled trial, anthocyanins showed anti-inflammatory effects by reducing hypercholesterolemic conditions in adults. This effect was consistent with previous observations as intake of anthocyanins significantly reduced LDL-cholesterol and HDL-cholesterol levels with inhibition of IL-6 and IL-1β in a HepG2 cell line [208]. It is noteworthy to observe that a mixture of anthocyanins was more effective than individual components for improved human health. As discussed earlier, it could be the anti-oxidant potential of flavonoids that might exert these effects, as observed from a recent cross-sectional study conducted in the Italian population against the human papillomavirus (hrHPV) infection. The study was conducted in 251 women with normal cervical cytology, showed a significant reduction in the risk of developing hrHPV infection with anthocyanins-rich diet [209]. Further, a flavonoid-rich diet has been studied for their protective effects against various disease conditions which are shown in Table 2.

Moreover, the dietary intake of flavonoids has a direct impact with microbiota as ingested flavonoids are known to be unabsorbed in the proximal intestine, further reach the colon region. This microenvironment facilitates the hydrolysis and fermentation of flavonoids employing different enzymes released by the microorganisms. Microbes can facilitate oxidation, demethylation, and catabolism of flavonoids into phenolic acids and aromatic catabolites [210]. Recent studies have shown that the high flavonoid-rich diet exerts an inverse correlation with obesity and inflammation. This is largely mediated by gut microbiota as it increases adiposity and fatty acid metabolism. Further, intestinal microbes modify flavonoids by glucosidation, dihydroxylation, and decarboxylation converting them into monomers, which can be more absorbable in the intestine [211]. To add, flavonoids isolated from mulberry leaves ameliorate lipid dysmetabolism in the high fat diet (HFD)-fed mice. Gut microbes such as *Bacteroidetes* played a role in the increased production of acetic acid, thereby restoring lipid metabolism [212]. From these observations, it is noteworthy to believe that both classifications of flavonoids and microbiota play a cross-talk between dietary intake and improved health benefits.

## 9. Conclusions and Future Directions

The role of p53 in inflammation and cancer is well established. Being a central protein in immune pathways, the modulation of p53 expression may lead to the prevention of many diseases. Further cross-talk between p53 and immune checkpoints, such as MHC1 and TLRs, has great potential in developing drugs, especially for cancer immunotherapy. As discussed in this review, flavonoids are one of the ideal choices of biomolecules for developing safer immunosuppresses in the management of chronic inflammation leading to cancer progression. Many of the flavonoids exert their preventive potentials by enhancing the endogenous anti-oxidant system through modulating the Keap1/Nrf2/ARE pathway. Although the p53-mediated Nrf2 pathway is not well studied, there is evidence to show that the activation of the Nrf2 pathway leads to a reduction in oxidative stress and enhanced DNA repair processes [223]. Thus flavonoids, especially with anti-oxidant properties, enhance the immune response against various oxidative stress-mediated cellular malfunctions leading to disease prevention. Further, a flavonoid-rich diet has shown evidence for the above cytoprotective mechanism in recent clinical trials, showing their disease-preventive potential.

However, direct links between flavonoid-mediated p53 and the anti-oxidant Keap1/Nrf2/ARE pathway to exert preventive potentials are scarce. In contrast, many reports have shown a p53- mediated induction of cell death pathways that can be regulated by flavonoids in various cancer models. Further, exploring the role of flavonoids through the p53 pathway in disease prevention should be of great interest for future research. Understanding the safe and efficacious dose of flavonoids is also very crucial in treatment since most flavonoids, depending on the concentration and microenvironment, can act as either anti-oxidants or prooxidants exhibiting hormetic effects. Moreover, continuous efforts are in progress for enhancing the bioavailability of flavonoids by acylation to enhance cellular uptake, improving stability by using different formulations, engineering microbiota for their improved impacts, and developing technologies, such as nanocrystals, for enhanced pharmacokinetics and targeted therapy.

## Figures and Tables

**Table 1:** Flavonoids in preventing inflammation and cancer in preclinical and experimental cell models.

Flavonoid	Disease/Oxidative Stress Imposed	Preclinical or Cell Model	Molecular Signaling/Pathway	Inflammation/Cancer Prevention Status	Reference
Apigenin	Cardiotoxicity	Rats	Decreases caspase-3 and Bax	Inhibition of apoptosis	[175]
	Myocardial injury	Mice	Inhibits TNF-α, IL-1β, MIP-1α, MIP-2, and NFκB	Inhibit inflammation	[176]
	UVB-induced skin cancer	Mice	Inhibits IL-6 and IL-12	Inhibit inflammation	[177]
	Bowel disease and colitis-associated cancer	Mice and HCT-116	Inhibits STAT3 and NF-κB	Inhibit inflammation-induced carcinogenesis	[178]
	Isoproterenol hydrochloride-induced apoptosis	H9C2	Inhibits Bax, caspase-3, -8, and -9 and cytochrome c	Cancer prevention	[179]
Chrysin	Cerebral ischemia	Rats	Inhibits IL-1β and TNF-α	Inhibit inflammation	[180]
	Cerebral ischemia	Rats	Inhibits TNF-α, IL-6, and IL-1β; activates PI3K/Akt/mTOR pathway	Inhibit inflammation and apoptosis	[181]
	Reproductive toxicity	Mice	Inhibits IL-1β, TNF-α, IL-6, and IL-10; activates caspase 3 and 9	Inhibit inflammation	[182]
	Hepatic encephalopathy	Rats	Increases glutathione level; inhibit NF-κB, TNF-α, IL-6, and TLR-4; reduces caspase-3 and hepatic necrosis	Inhibit inflammation and apoptosis	[183]
Daidzein	Endometriosis	OESCs and NESCs	Inhibited IL-6, IL-8, COX-2, TNF-α-induced IκB phosphorylation and p65	Inhibit inflammation	[184]
	Intestinal mucositis	Mice	Inhibits TNF-α, IL-1β, and IL-6; increases CAT and GPx level	Inhibit inflammation	[185]
Naringenin	Obesity	Mice	Inhibits TLRs and TNF-α	Inhibit inflammation	[186]
	Pulmonary metastasis	Mice	Inhibits Tgf-β1	Cancer prevention	[187]
	Cardiac hypercholesterolemia	Rats	Inhibits DNA damage, TNF-α, and RIP3	Inhibits necroptosis	[188]
Kaempferol	Propacetamol-induced acute liver injury	Mice	Inhibits cytochrome P450 2E1; restores SOD, GPx and CAT; inhibits Bax/Bcl-2 ratio	Prevents apoptosis	[189]
	Etoposide-induced oxidative stress	HL-60 and PBMCs	Inhibits DNA damage (tail length)	Prevents DNA damage	[190]
	Paw Edema	Mice and THP-1 (together)	Inhibits Cox-2; STAT3 and NF-κB	Inhibit inflammation	[191]
	Brain Injury and neuroinflammation	Rats	Inhibits STAT3 and NF-κB p65	Inhibit inflammation	[189]
Fisetin	Hypoxia	NCI-H157	Inhibit HIF 1-α and STAT3	Prevents hypoxia	[192]
	Hydrogen peroxide-induced oxidative stress	HaCaT	Inhibits iNOS, Cox-2, IL-1β, IL-6, and TNF-α	Inhibit inflammation	[193]
	Diabetic cardiomyopathy	Rats	Restores SOD and CAT; inhibits IL-1β, IL-6, and TNF-α; inhibits caspase-3, Bax, and Bax/Bcl-2 ratio	Inhibit inflammation and apoptosis	[194]
Myricetin	LPS-induced Inflammation	Mice and RAW 264.7	Inhibits NF-κB p65 and AKT activation	Inhibit inflammation	[195]
	Colonic chronic-induced inflammation	Mice	Inhibits TNF-α, IL-1β, IL-6, NF-κB, p-NF-κB, cyclooxygenase-2 (COX-2), PCNA, and Cyclin D1	Inhibits inflammation and tumor	[196]
Hesperetin	Hepatic fibrosis	Mice and HSCs	Inhibit α-SMA, Col1α1, Col3α1 and TIMP-1	Inhibits inflammation and induce apoptosis	[197]
	Testicular Damage	Rats	Inhibits malondialdehyde, ROS, DNA fragmentation, and caspase 3; inhibits TNFα and IL-17	Inhibits inflammation and apoptosis	[198]
Catechin	TNF-α-induced inflammation	3T3-L1	Inhibit IL-1α, IL-1β, IL-6, IL-12, p35, iNOS, Cox-2, NF-κB, AMPK, FOXO3a, and SIRT1	Inhibits inflammation	[199]
	Methylglyoxal-induced cytotoxicity	EA.hy926	Inhibits MMP and cytotoxicity	Inhibits apoptosis	[200]

**Table 2:** Flavonoids-rich diet in disease prevention: a summary from recent (2019–2020) clinical trials.

Dietary Flavonoids	Condition Studied	Sample Details	Key Observations	Reference
*Seidlitzia Rosmarinus* (flavonoid-rich)	Recurrent cystitis	126 women	Lowered symptoms of cystitis; prevent the incidence of recurrent cystitis with no side effects	[213]
Genistein with FOLFOX	Metastatic colorectal cancer	13 patients	Exposure of genistein with FOLFOX was safe and tolerable	[214]
Anthocyanin-rich blueberry	Type 2 diabetes and CVD	115 people	Improved endothelial function, systemic arterial stiffness and attenuated cyclic guanosine monophosphate concentrations	[215]
Anthocyanin-rich fruit juice	Healthy volunteers	57 males	Demonstrated DNA-protective and anti-oxidant effects; reduction in body fat and an increase in fat-free mass with increased SOD level	[216]
Flavonoid-rich natural cocoa beverage	Plasma oxidative stress and inflammation	134 people	Improved glycemia, triglyceridemia, high-density lipoprotein cholesterol, low-density lipoprotein cholesterol, triglyceridemia/HDL index, and oxidative markers	[217]
Genistein	Human prostate cancer	14 males	Increased Brain Abundant Membrane Attached Signal Protein 1(BASP1) expression; decreases MMP-2 in prostate tissue	[218]
EGCG	Acute radiation-induced esophagitis (ARIE)	83 patients	Lowered ARIE by reducing acute pain index (API) and acute dysphagia index (ADI) without side effects	[219]
EGCG	Cutaneous scarring	62 humans	Reduced mast cells; down-regulated Vascular Endothelial Growth Factor A (VEGFA) and CD31; reduced scar thickness	[220]
Quercetin	Eccentric exercise-induced muscle damage	12 males	Increased the isometric strength for contraction; lower torque and muscle fiber conduction velocity; attenuate the severity of muscle weakness	[221]
Silymarin	Radiation-induced dermatitis	40 patients	Delayed in radiodermatitis development and progression	[222]

## Abbreviations

AID	activation-induced cytidine deaminase
AOM	azoxymethane
AP1	activator protein *1*
ARE	anti-oxidant response element
ATP	adenosine triphosphate
BSO	buthionine sulfoximine
Cag-A	cytotoxin-associated gene A
Cat	catalase
CIA	collagen-induced arthritis
CNS	central nervous system
CVD	cardiovascular diseases
DDR	DNA damage **r**esponse
DMH	1,2-dimethylhydrazine
EAE	experimental autoimmune encephalomyelitis
EGCG	epigallocatechin gallate
ERAP	endoplasmic reticulum aminopeptidase 1
HCC	hepatocellular carcinoma
HFD	high fat diet
HIF1	hypoxia inducible factor
IKK	IκB kinase
IPM	immune prognostic model
LFS	Li-Fraumeni syndrome
LOH	loss of heterozygosity
LPS	lipopolysaccharides
M-CSF	macrophage colony-stimulating factor
MHC	major histocompatibility complex
MMP	matrix metalloprotease
NF-κB	nuclear factor-κB
NRF2	nuclear factor erythroid 2-related factor 2
PAMPs	pathogen-associated molecular patterns
PPAR	peroxisome proliferator-activated receptors
PTEN	phosphatase and tensin homolog
ROS	Reactive oxygen species
SOCS1	suppressors of cytokine signaling 1
SOD	superoxide dismutase
STAT3	signal transducer and activator of transcription 3
TAM	tumor-associated macrophage
TAP1	transporter associated with Antigen Processing 1
TLR	toll-like receptor
TNBS	2,4,6-trinitrobenzenesulfonic acid
UVB	ultraviolet B radiation
WT	wild type
